# Study on the different responses of different winter wheat cultivars to dry hot wind

**DOI:** 10.1371/journal.pone.0274118

**Published:** 2022-10-05

**Authors:** Xiyan Kang, Zhangyan Le, Chunqiang Li, Liqin Dai, Chang Quan, Minghua Shi, Rongwei Liao

**Affiliations:** 1 Hebei Provincial Key Laboratory of Meteorological and Eco-environment, Shijiazhuang, China; 2 Meteorological Science Institute of Hebei Province, Shijiazhuang, China; 3 Langfang Meteorological Bureau of Hebei Province, Langfang, China; 4 State Key Laboratory of Severe Weather, Chinese Academy of Meteorological Sciences, Beijing, China; China National Rice Research Institute (CNRRI), CHINA

## Abstract

Dry hot wind (DHW) is one of the main agro-meteorological disasters that occur during the grain filling stage of winter wheat in northern China. In this study, three major winter wheat cultivars planted at the Mazhuang experimental station, Xinji city, Hebei Province, including Henong 6119 (HN6119), Gaoyou 5218 (GY5218), and Jimai 325 (JM325), were analyzed. Through natural DHW and artificially simulated DHW experiments, we investigated how the physiological parameters of the three cultivars were affected on the day with DHW and the day before and after DHW occurred. Comparative analysis of the different responses among the physiological parameters of the three cultivars demonstrated that HN6119 experienced less leaf water loss by reducing its stomata conductance and transpiration rate under natural DHW conditions, while GY5218 and JM325 experienced more leaf water loss by increasing their stomata conductance and transpiration rates under natural DHW conditions. The net photosynthetic rate, transpiration rate, and stomata conductance of HN6119 recovered after the DHW conditions, while those of GY5218 and JM325 showed a continuously decreasing trend. The leaf photosynthetic water use efficiency decreased on DHW days because the net photosynthesis rate was reduced for HN6119, but the transpiration rate increased for GY5218 and JM325. HN6119 showed a significant positive correlation between physiological parameters, while GY5218 and JM325 showed a poor correlation after being affected by DHW conditions. The effect of artificial simulation under mild and severe DHW stress on the thousand kernel weight (TKW) of HN6119, GY5218 and JM325 was 0.01%, 3.51%, 3.57% and 0.36%, 8.12%, 8.84%, respectively. HN6119 showed better resistance to DHW, followed by GY5218, and JM325 showed the weakest resistance.

## Introduction

Dry hot wind (DHW) is a kind of catastrophic weather with a high temperature, low relative humidity and high wind speed, and this type of weather has different names across many countries, such as “sharav” in Israel, “sirocco” in North Africa, and “sukhovei” in the former Soviet Union [1]. It is one of the main agro-meteorological hazards that occurs during the grain filling stage of winter wheat in North China and can cause wheat yield reductions of 10% to 20% under severe DHW weather [2,3]. Hebei Province is one of the main wheat planting areas in China and a region with severe DHW damage [4], and the winter wheat area of central and southern Hebei are DHW high-risk zones [5]. Under the conditions of global warming, the frequency and severity of extreme weather and climate events, such as droughts and high temperatures, are likely to increase in the future [6–15]. Zhao et al. reported that the most serious damage caused by light and severe DHW over the past 50 years (1961–2010) occurred in the 1960s and then in the 1970s and 2000s on the Huang-Huai-Hai Plain in China [16]. Therefore, studying the responses of different winter wheat cultivars to DHW is of great practical significance for understanding the characteristics of different winter wheat cultivars and the production of winter wheat.

Many researchers have paid substantial attention to studying the spatial-temporal characteristics of DHW and its impact on crop physiological functions and yields. Lydolph noted the index of sukhvoey for crop reactions and methods of reducing its negative effects in Russia and its distribution and two cases in North America [17,18]. Tavakol et al. [19] analyzed the spatial temporal variations in the frequency of compound DHW events in the central United States. Smika et al. [1] reported the effect of DHW on wheat growth and yield by means of a wind tunnel test and found that DHW reduced the number of spikes, grain number per spike and grain weight of winter wheat. Studies on the DHW hazards for winter and spring wheat in China began in the late 1950s, and the meteorological index, damage mechanism, effects on crop growth and yield, and defense measures of DHW have been studied over the last 40 years [20–26]. Some researchers have discussed the different resistances of different wheat cultivars to DHW [27,28]. The effects of DHW on the photosynthetic physiological parameters of wheat were studied by artificially simulated DHW experiments after 2000, and some experiments were combined with remote sensing models to study the effects of DHW on the stomatal behavior of winter wheat [29–32]. Most previous studies focused on the comparison of photosynthetic physiological parameters before and after DHW event, while there are few studies on the variation characteristics of each parameter on the day affected by DHW. Additionally, most of the studies were carried out by using a single winter wheat variety. With continually updated winter wheat cultivars, it is essential to investigate the different responses of different winter wheat cultivars to DHW to meet the needs of modern agricultural production.

In this study, the experiment was carried out at the Mazhuang (37°58′N, 115°13E′) experimental station of Hebei Agricultural University in Xinji city, Hebei Province, China, during 2019–2020. The terrain is flat, and the station is surrounded by farmland. The climate is warm temperate with subhumid lands, an annual average temperature of 13.6°C, an annual rainfall amount of 466.4 mm mainly from June to September, an annual relative humidity of 63%, and annual sunshine of 2610.1 h. The soil type is loamy with a pH of 7.1. The soil has medium fertility and an average field capacity of 22.7% (percentage of dry soil quality), a bulk density of 1.37 g cm^-3^, and an average wilting humidity of 5% (percentage of dry soil quality). The objective of this study was to identify the response characteristics of the three winter wheat cultivars to DHW in Hebei Province. Under natural and artificially simulated DHW conditions, we conducted field experiments in 2019 and 2020 to examine the responses of winter wheat physiological characteristics, such as net photosynthetic rate (P_n_), transpiration rate (T_r_), and stomatal conductance (G_s_), to identify the effects of DHW on leaf photosynthetic water use efficiency and thousand kernel weight (TKW) of wheat and to provide useful information for winter wheat production and cultivar choice.

## Materials and methods

### Experimental materials and design

The experiment was carried out in one of the main wheat planting regions of Hebei Province. In addition, the phenotypic differences among varieties (including plant height, days of full growth period, panicle type, etc.) were also considered. The three tested winter wheat cultivars were Henong 6119 (HN6119), Gaoyou 5218 (GY5218), and Jimai 325 (JM325); the plant heights were 70.7 cm, 72.71 cm and 75 cm, respectively, and the days of the whole growth period were 239 days, 240 days and 242 days, respectively. The panicle types were spindle shaped, rectangular and nearly rectangular. There were 6 planting plots in this experiment for the three different cultivars, and the same cultivar was planted in every two plots. The planting plot of each variety was 2000 cm × 3000 cm in size, and each variety contained a replicate area with the same sowing time. Fertilizers (Shidanli compound fertilizer) were manually broadcasted before sowing and incorporated during basal application at rates of 389 kg ha^-1^ N, 75 kg ha^-1^ K and 55 kg ha^-1^ P. Fertilization, irrigation, pest control and other management practices were consistent across plots. The initial flowering date of the three tested wheat was May 3^rd^, 2019, and there were no plant diseases or insect pests and no drought during the whole growth period. The categorization of DHW was performed according to Huo et al. [33] (shown in Table 1).

**Table 1:** Classification of dry hot wind.

Class	Daily Max.Tem. (°C)	Relative Humidity (%)*	Wind speed(m/s)*
mild	≥32	≤30	≥3
severe	≥35	≤25	≥3

*: Relative humidity and wind speed at 14 pm.

A mild DHW occurred on May 22^nd^, 2019, from 13:00 to 18:00, and a severe DHW occurred on May 23^rd^, 2019, from 13:00 to 17:00. The field measurements were carried out from May 21^st^ to May 24^th^ and on May 28^th^ and May 31^st^ 2019, when the weather conditions were good. Table 2 gives the meteorological element values on DHW days in 2019.

**Table 2:** Hourly meteorological data from 10:00–19:00 on May 22^nd^ and 23^rd^, 2019.

Time		10:00	11:00	12:00	13:00	14:00	15:00	16:00	17:00	18:00	19:00
5/22	Temperature (°C)	28.1	30.1	32.3	32.4	33.6	34.4	33.2	34.7	32.8	28.6
	Wind speed (m/s)	1.2	1.5	2.1	3.3	3.0	3.0	3.8	3.0	3.0	3.2
	Relative humidity(%)	26	18	16	16	18	17	16	17	21	26
5/23	Temperature (°C)	29.6	32.0	34.3	34.0	35.7	37.1	34.9	34.6	31.9	30.6
	Wind speed (m/s)	1.1	0.7	1.7	3.0	3.0	3.0	3.5	3.0	3.2	3.8
	Relative humidity(%)	26	23	21	15	17	13	13	13	15	17

In 2020, no DHW weather occurred during our experiment; hence, we used the DHW simulation generator developed by the Institute of Geographic Sciences and Natural Resources Research of the Chinese Academy of Sciences to conduct the experiment and a control test (i.e., normal growth and not exposed to DHW), which were designed during the experiment at the same time. Specifically, a mild DHW experiment was carried out from 13:00 to 16:00 on May 23^rd^, with an average temperature of 35.8°C, a relative humidity of 26.9% and a wind speed of 3 m/s. Moreover, we carried out observations on May 23^rd^ (the DHW day), May 24^th^ (the day after the DHW day), and May 26^th^ (three days after the DHW day) 2020. In addition, a severe DHW experiment was carried out from 10:00 to 17:00 on May 24^th^, with an average temperature of 39.5°C, a relative humidity of 20.8%, and a wind speed of 3 m/s. Moreover, we carried out observations on May 24^th^ (the DHW day), May 25^th^ (the day after the DHW day) and May 27^th^ (three days after the DHW day). After the wheat matured in 2020, the TKW, grain length, grain width and grain thickness of the different wheat cultivars were investigated.

The experiment conducted complied with relevant institutional, national, and international guidelines and was approved and guided by the Ethics Committee of the China Meteorological Administration. This study obtained permission to collect the wheat cultivars Henong 6119 (HN6119), Gaoyou 5218 (GY5218), and Jimai 325 (JM325) during the experiment in China.

### Measurement and methods

The net photosynthetic rate (P_n_), transpiration rate (T_r_) and stomatal conductance (G_s_) were measured by the LI-6400XT Portable Photosynthesis System (LI-COR Inc, United States). As the flag leaf is the primary photosynthetic organ for grain filling and yield formation [34], 5 flag leaves with a similar growth pattern, which were designed to repeat observation 5 times, were selected randomly from each cultivar for the physiological parameter measurements, and each leaf was measured 3 times. Therefore, 15 data points were acquired in one observation. We excluded the data with the largest deviation and considered the average value of the remaining 14 data points as the value in one observation. To avoid the influence of the daily variation in different parameters [35–40] on the comparability, the measurement was at 14:00 pm in 2019 (intensive observations at 10:00 am, 12:00 pm, 16:00 pm and 18:00 pm on DHW days) and immediately after the DHW stress experiment in 2020, with 3 replications for three winter wheat cultivars. After the measurements, five leaves were cut and weighed to determine their fresh weight (FW) and then weighed to determine their dry weight (DW) after treatment at 120°C for 20 minutes and drying at 80°C for 10 hours in an oven. Therefore, the relative water content (RWC) of the leaves was calculated by the following formula:

$$RWC=\frac{FW-DW}{FW}\times 100\%$$ (1)

The stress index (SI) of DHW on the parameters was calculated as follows:

$$SI=\frac{\left|n_{b}-n_{a}\right|}{n_{a}}\times 100\%$$ (2)

where *n*_*b*_ is the value measured for physiological parameters (i.e., P_n_, T_r_, and G_s_) after DHW stress, *n*_*a*_ is the value measured for physiological parameters (i.e., P_n_, T_r_, and G_s_) without DHW stress; if *n*_*b*_>*n*_*a*_, then it indicates that there is no obvious negative effect on wheat after DHW stress. When the *SI* value is larger, the disaster stress is stronger, and vice versa.

The *F* test and correlation method were used to analyze the responses of the different winter wheat cultivars to DHW. Table 3 shows the significance test results of the photosynthetic physiological parameters measured in 2019.

**Table 3:** Significance test of photosynthetic physiological parameters for the three cultivars during the 2019 experiment.

Date	HN6119			GY5218			JM325		
	P_n_	T_r_	G_s_	P_n_	T_r_	G_s_	P_n_	T_r_	G_s_
5/21–5/22	**	ns	**	ns	*	ns	ns	**	ns
5/22–5/23	ns	ns	ns	ns	**	**	**	**	**
5/23–5/24	ns	**	ns	*	**	**	*	**	ns
5/24–5/28	*	ns	**	**	ns	**	**	**	**
5/28–5/31	**	**	**	**	**	**	**	**	**

Note: ns

*, ** indicate that F tests are not significant, P<0.05 and P<0.01 respectively.

## Results

### Differences in the responses of net photosynthetic rate (P_n_) for the three cultivars

**Fig 1:**
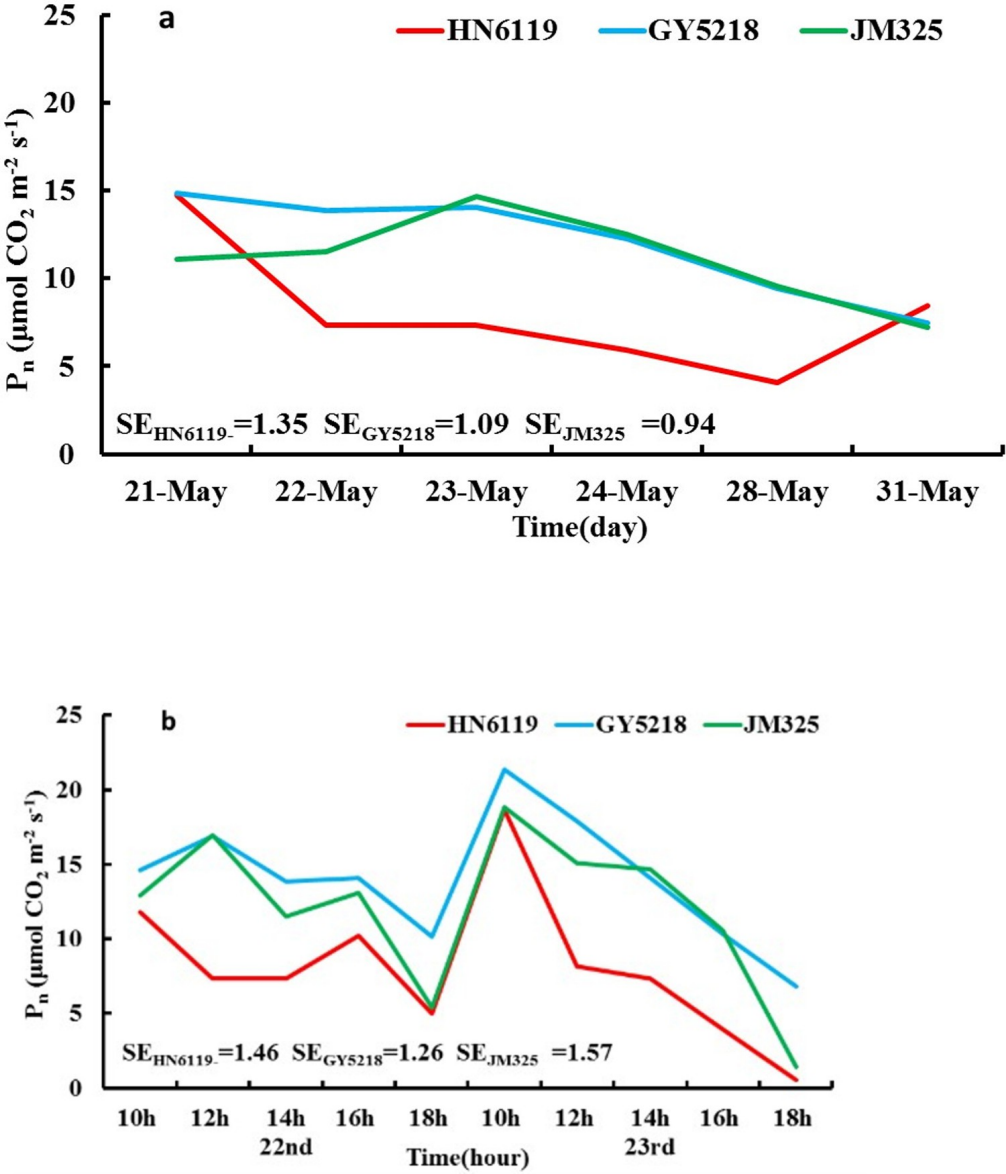
(a) Change curves of P_n_ (unit: μmol CO_2_ m^-2^ s^-1^) for the three cultivars affected by DHW from May 21^st^ to 24^th^ and on May 28^th^ and May 31^st^, 2019; (b) the daily change curves of P_n_ (unit: μmol CO_2_ m^-2^ s^-1^) for the three cultivars affected by DHW from May 22^nd^ to 23^rd^, 2019. (SE is the abbreviation of the standard error of P_n_. The red, blue, and green lines denote the wheat cultivars for HN6119, GY5218, and JM 325, respectively).

Fig 1A presents the change curve of P_n_ (μmol CO_2_ m^-2^ s^-1^) for each cultivar from May 21^st^ to 24^th^ and on May 28^th^ and May 31^st^ in 2019, and the P_n_ of HN6119 showed a decreasing trend from May 21^st^ to May 24^th^ and an increasing trend on May 28^th^ and May 31^st^. The *F* test result for HN6119 was extremely significant from May 21^st^ to May 22^nd^ and May 28^th^ to May 31^st^ and significant from May 24^th^ to May 28^th^. The P_n_ of GY5218 showed no significant change from May 21^st^ to May 23^rd^, a significant decrease from May 23^rd^ to May 24^th^, and an extremely significant decrease on May 24^th^ and May 31^st^. The P_n_ of JM325 showed an increasing trend from May 21^st^ to May 23^rd^ and was similar to that of GY5218 after May 23^rd^. The *F* test result for JM325 was extremely significant from May 22^nd^ to May 23^rd^.

Under the influence of DHW, the variation characteristics of P_n_ showed differences among cultivars, which indicated that the responses of different cultivars to DHW were not the same. For HN6119, the P_n_ was at a high level on May 21^st^, and it decreased from May 22^nd^ to 23^rd^ when DHW occurred. While it showed a significant increasing trend on May 28^th^ and May 31^st^ during the recovery time after DHW conditions, DHW had little effect on it. For GY5218, the P_n_ on May 21^st^ was also at a high level; it showed no significant change and remained at a high level when DHW occurred, and there was a significant decreasing trend and no sign of recovery of P_n_ when the DHW ended, which indicated that DHW had a negative effect on it. For JM325, the crop responded to it by increasing the P_n_ when DHW occurred, and there were no signs of recovery of P_n_ but with a continuous decreasing trend when DHW ended, which indicated that DHW also had effects on it.

Fig 1B is the daily change curve of P_n_ on the day when DHW occurs. For HN6119, the daily change curve of P_n_ showed a bimodal pattern on May 22^nd^, 2019, with two peaks at 10:00 am and 16:00 pm, which indicated that mild DHW had no obvious effect on this cultivar. This result was similar to the observation shown in previous literature [29]. On May 23^rd^, P_n_ still reached a higher level at 10:00 am, but this peak was followed by a continuous decrease when the temperature increased, as shown in Table 2; in addition, no peak appeared at 16:00, which indicated that severe DHW had a significant effect on this cultivar. For GY5218 and JM325, the peak of P_n_ was observed at 12:00 pm on May 22^nd^, and the peak time showed a significant difference from that in a previous study [29]. This result indicated that mild DHW had a certain but not severe effect on these cultivars. On May 23^rd^, P_n_ also reached a higher level at 10:00 am, and the change was similar to that for HN6119 afterward, which indicated that severe DHW caused damage to these two cultivars.

The P_n_ of HN6119 was sensitive to DHW, and its P_n_ decreased greatly; however, the P_n_ of GY5218 and JM325 was not sensitive to DHW; therefore, their P_n_ had a minimal decrease.

### Differences in the responses of the transpiration rate (T_r_) for the three cultivars

**Fig 2:**
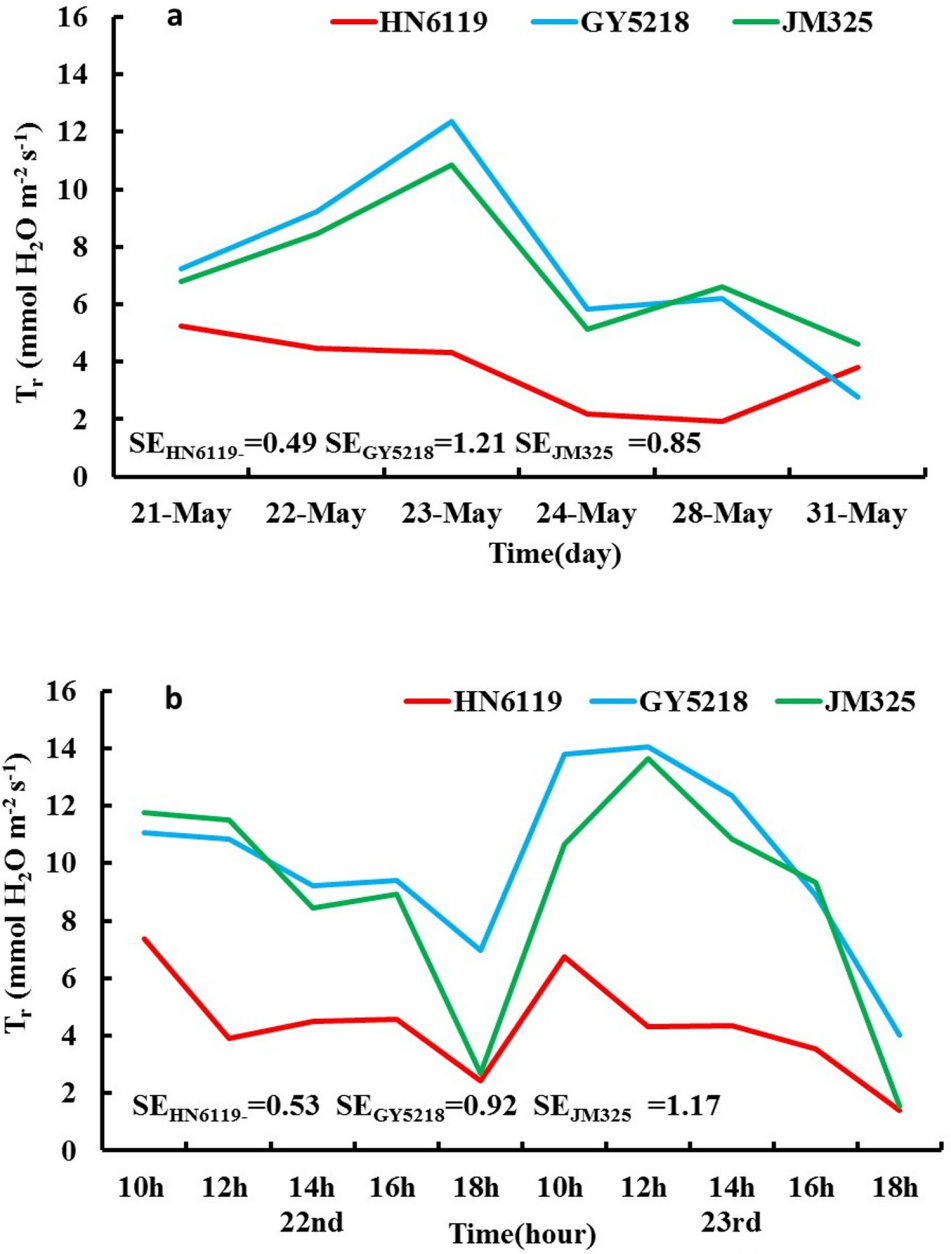
As in Fig 1, but for (a) T_r_ (unit: mmol H_2_O m^-2^ s^-1^) from May 21^st^ to 24^th^ and on May 28^th^ and May 31^st^, 2019; (b) T_r_ (unit: mmol H_2_O m^-2^ s^-1^) from May 22^nd^ to 23^rd^, 2019. (SE is the abbreviation of the standard error of T_r_).

Fig 2A is the T_r_ (mmol H_2_O m^-2^ s^-1^) change curve of the three winter wheat cultivars from May 21^st^ to 24^th^ and on May 28^th^ and May 31^st^, 2019. As shown in Fig 2A and Table 3, the T_r_ of HN6119 showed a decreasing trend from May 21^st^ to May 24^th^ and an increasing trend on May 28^th^ and May 31^st^. The *F* test of T_r_ for HN6119 was extremely significant from May 23^rd^ to May 24^th^ and on May 28^th^ and May 31^st^. The T_r_ change trends of GY5218 and JM325 were similar; both showed a continuous increase from May 21^st^ to May 23^rd^, while they significantly decreased from May 23^rd^ to May 24^th^ and on May 28^th^ and May 31^st^. The increasing trends of GY5218 and JM325 from May 21^st^ to May 23^rd^ were significantly different, but the decreasing trend was almost the same; the overall change trend decreased after May 23^rd^ 2019.

The different variation characteristics of T_r_ indicated that the responses of different cultivars to DHW were different. For HN6119, the variation characteristics of T_r_ were similar to those of P_n_, and the crop responded to DHW by decreasing the T_r_, which indicated that DHW had little effect on it. For GY5218 and JM325, the crop responded to DHW by increasing the T_r_, but T_r_ showed a significant decreasing trend when DHW ended, which indicated that DHW had an effect on it. The value of T_r_ was closely related to the amount of water loss in leaves.

Fig 2B shows the T_r_ daily variation curve of the three cultivars experiencing DHW on May 22^nd^ and 23^rd^ 2019. The T_r_ remained at a low level at the beginning and continuously decreased on the day with severe DHW for HN6119. The T_r_ remained at a high level at the beginning and peaked at 12:00 pm on May 23^rd^ for GY5218 and JM325. The results showed that the T_r_ of HN6119 decreased to reduce the water loss from the leaves, while the T_r_ of GY5218 and JM325 increased to increase the water loss from the leaves when they were affected by DHW. From Table 4, it was obvious that the variations in the leaves’ FWs, DWs and RWC of the three cultivars occurred one day before (May 21^st^) and after (May 24^th^) DHW. The RWC of the leaves decreased by 0.24%, 1.94%, and 3.71% for HN6119, GY5218, and JM325, respectively (Table 4). This result indicated that the T_r_ of HN6119 decreased and caused less water loss from the leaves, while the T_r_ of GY5218 and JM325 increased and caused more water loss from the leaves.

**Table 4:** FW (unit: g), DW (unit: g), RWC (unit: %) and standard error (unit: g) of the leaves of the three cultivars on the day before and after the DHW day during the 2019 experiment.

	HN6119			GY5218			JM325		
Date	FW	DW	RWC	FW	DW	RWC	FW	DW	RWC
5/21	1.041	0.346	66.77	1.413	0.463	67.22	2.867	0.818	71.46
5/24	0.899	0.300	66.61	1.000	0.341	65.92	2.054	0.641	68.81
Standard Error	0.050	0.016	0.057	0.146	0.043	0.460	0.287	0.063	0.937
Reduction (%)	13.6	13.2	0.24	29.2	26.4	1.94	28.4	21.7	3.71

The T_r_ variations among the three cultivars implied that HN6119 with the low T_r_ was not very sensitive to DHW, but GY5218 and JM325 with the high T_r_ were sensitive to DHW; thus, their T_r_ had greatly changed.

### Differences in the responses of the stomatal conductance (G_s_) for the three cultivars

**Fig 3:**
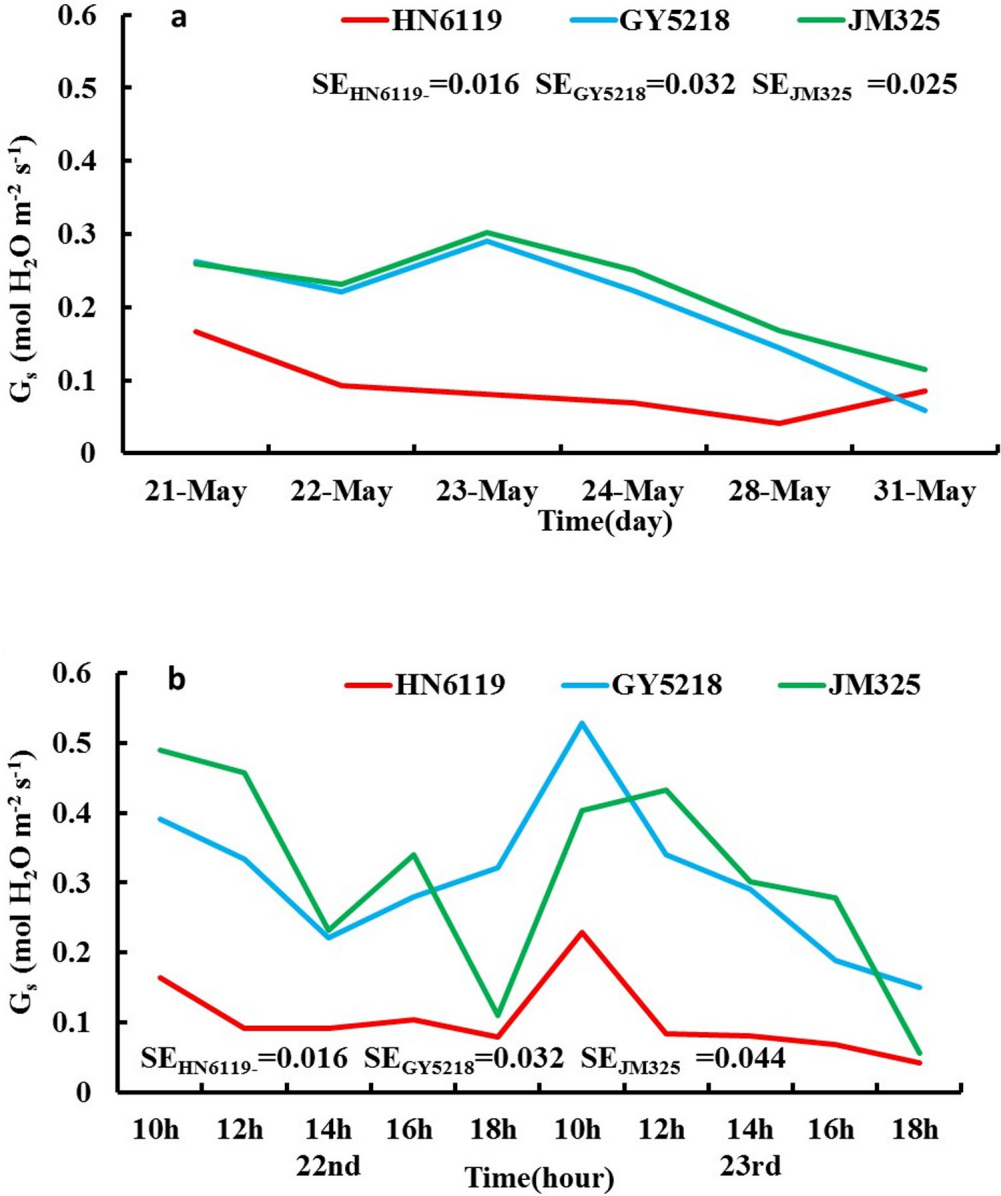
As in Fig 1, but for (a) G_s_ (unit: mol H_2_O m^-2^ s^-1^) from May 21^st^ to 24^th^ and on May 28^th^ and May 31^st^, 2019; (b) G_s_ (unit: mol H_2_O m^-2^ s^-1^) from May 22^nd^ to 23^rd^, 2019. (SE is the abbreviation of the standard error of G_s_).

Fig 3A shows the G_s_ (mol H_2_O m^-2^ s^-1^) change in the three winter wheat cultivars from May 21^st^ to 24^th^ and on May 28^th^ and May 31^st^. The G_s_ variation tendency of the three cultivars was significantly different. For HN6119, G_s_ showed a decreasing trend from May 21^st^ to May 24^th^ and an increasing trend on May 28^th^ and May 31^st^. The variation characteristics were similar to those of P_n_ and T_r_, and significant changes were observed on May 21^st^, 22^nd^, May 24^th^, May 28^th^, May 28^th^ and May 31^st^. For GY5218 and JM325, the G_s_ variation characteristics were similar; both showed a significant increasing trend from May 22^nd^ to May 23^rd^ and a decreasing trend after May 23^rd^. However, a significant decrease was found in different periods for GY5218 and JM325, as shown in Table 3.

The variation characteristics of G_s_ showed the different responses by the three cultivars to DHW. For HN6119, the G_s_ decreased during the DHW conditions and recovered after the DHW, which indicated that DHW had little effect on it. GY5218 and JM325 responded to DHW by increasing G_s_, with a continuous decreasing trend and no signs of recovery when DHW ended, which indicated that DHW had an effect on them.

From Fig 3B, it can be seen that G_s_ of HN6119 remained at a low level from the beginning, which was similar to the variation characteristic of P_n_ and T_r_, and HN6119 showed strong resistance to DHW by reducing the stomatal conductance to reduce T_r_ and P_n_ to reduce the damage caused by dry hot wind. For GY5218 and JM325, G_s_ remained at a high level when affected by DHW, which was consistent with the variation characteristic of T_r_. However, the increase in G_s_ and T_r_ at 12:00 pm, 14:00 pm, and 16:00 pm on the day affected by severe DHW was significant compared with the increase in P_n_. This result indicated that the increase in T_r_ did not cause the response of P_n_ and only increased the water loss of leaves, so the resistance to DHW of GY5218 and JM325 was weak. HN6119 had low G_s_ and was not sensitive to DHW, while GY5218 and JM325 had high G_s_ and were sensitive to DHW, so their G_s_ had greatly changed.

### Differences in leaf photosynthetic water use efficiency (WUE) among the three cultivars

**Fig 4:**
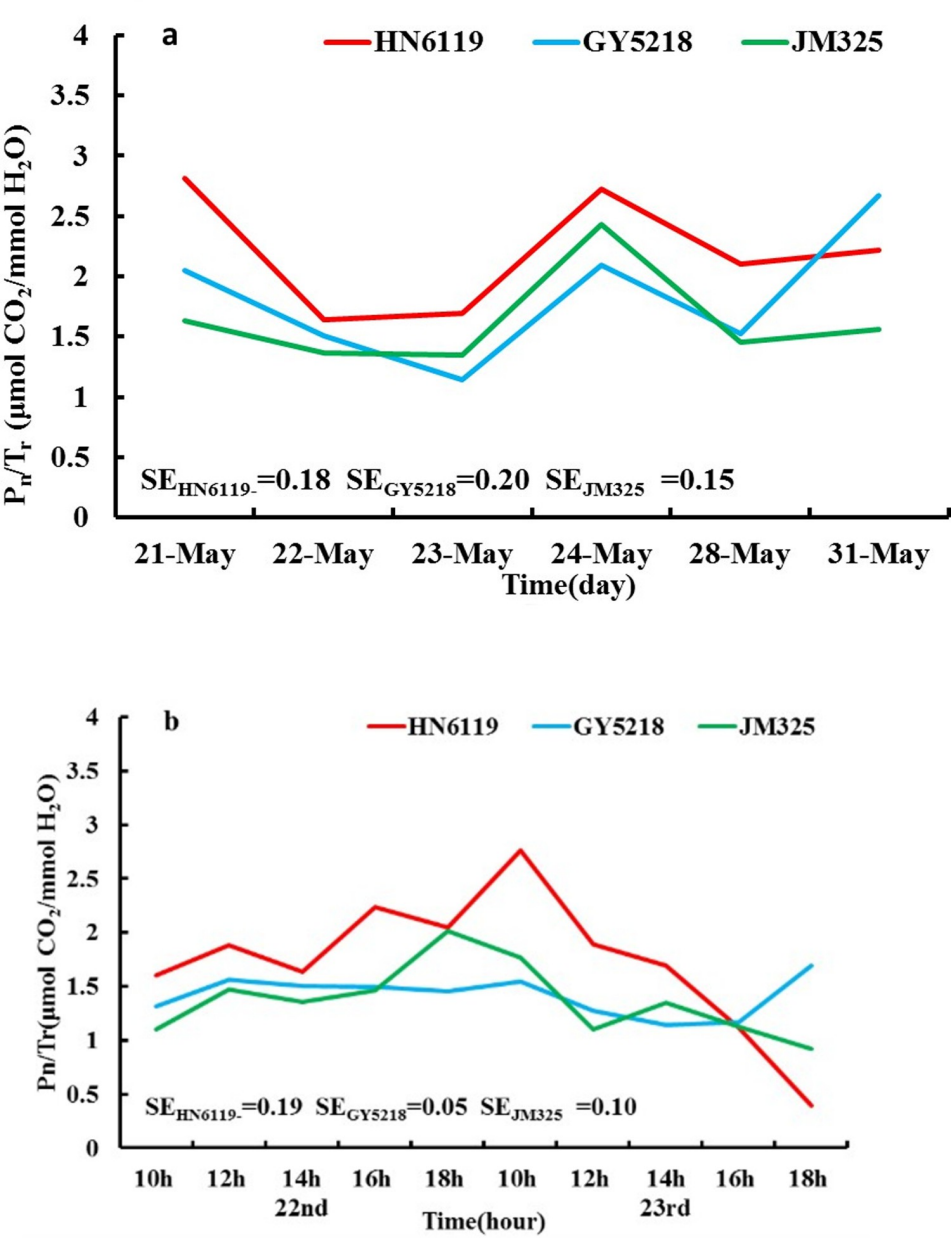
As in Fig 1, but for (a) leaf photosynthetic water use efficiency (WUE) (unit: μmol CO_2_/mmol H_2_O) from May 21^st^ to 24^th^ and on May 28^th^ and May 31^st^, 2019; (b) leaf photosynthetic WUE (unit: μmol CO_2_/mmol H_2_O) from May 22^nd^ to 23^rd^, 2019. (SE is the abbreviation of the standard error of P_n_/T_r_).

Fig 4A shows the variations in leaf photosynthetic water use efficiency (WUE), i.e., the ratio of the flag leaf’s net photosynthetic rate to transpiration rate, for the three cultivars from May 21^st^ to May 24^th^ and on May 28^th^ and 31^st^. The WUE of the three cultivars decreased under DHW conditions from May 22^nd^ to 23^rd^ and increased after DHW. The reason for the WUE decrease was not the same among the three cultivars. Generally, for HN6119, the net photosynthetic rate decreased, and the transpiration rate decreased minimally; however, for GY5218 and JM325, the transpiration rate increased, and the net photosynthetic rate changed minimally. After the DHW conditions, the WUE of the three cultivars recovered gradually to some extent on May 28^th^ and 31^st^, indicating that the three cultivars resisted DHW by changing WUE by adjusting plant physiological processes. As shown in Fig 4B, the WUE of HN6119 was higher than those of GY5218 and JM325 on DHW days. Under mild DHW conditions, the WUE changed for the three cultivars in two kinds of patterns: one had two peaks for HN6119, and the other had one peak for GY5218 and JM325. Under severe DHW conditions, the changes in WUE for the three cultivars showed almost the same decreasing trends, implying that the differences in the WUE variations for the three cultivars were not the same for the different intensities of DHW.

### Differences in the responses of the photosynthesis physiological parameters for the three cultivars

To examine the different responses of each cultivar to DHW, the correlation coefficients of the photosynthetic physiological parameters of the three cultivars were analyzed.

As shown in Table 5, the correlation coefficients of T_r_ and G_s_ of the three cultivars [41] for all measurements showed a significant positive correlation (*P* < 0.001); the correlation coefficients of P_n_, T_r_ and G_s_ before and after DHW days were also significantly positively correlated (*P* < 0.001). The significance levels of the correlation coefficients of P_n_, T_r_, and G_s_ on DHW days were different among the three cultivars. The correlation of all measurements was significantly positive at *P* < 0.001 for HN6119 and at *P* < 0.05 for GY5218. For JM325, the correlation coefficients were significantly positive except for the correlation between P_n_ and T_r_ at 16:00 pm and 18:00 pm on May 22^nd^ and the correlations of P_n_, T_r_, and G_s_ at 14:00 pm and 16:00 pm on May 23^rd^, which were closely related to the effect of DHW; however, some parameters had no significant correlation. Based on the hourly meteorological elements, the DHW lasted from 13:00 pm to 18:00 pm on May 22^nd^ and lasted from 13:00 pm to 17:00 pm on May 23^rd^. The correlation of P_n_ and T_r_ of JM325 was affected by DHW at 16:00 pm on May 22^nd^ and continued to be affected until May 23^rd^. The effect on the correlation of P_n_ and T_r_ was exacerbated when a severe DHW occurred as the temperature reached 35.7°C at 14:00 pm and 37.1°C at 15:00 pm on May 23^rd^, affecting the correlation of P_n_ and G_s_. The correlation difference in these parameters suggested that HN6119 had a better self-stability when affected by DHW, and its metabolism balance still maintained normal regulation. For GY5218 and JM325, the correlation coefficients of P_n_, T_r_ and G_s_ showed poor correlations when affected by DHW, which indicated that their regulatory function had been disrupted.

**Table 5:** Correlation coefficient of different photosynthetic physiological parameters for the three cultivars from May 21^st^ to 24^th^ during the 2019 experiment.

DATE		HN6119		GY5218		JM325	
		P_n_	T_r_	P_n_	T_r_	P_n_	T_r_
5/21	T_r_	0.912***		0.781***		0.905***	
	Gs	0.891***	0.957***	0.862***	0.964***	0.874***	0.993***
5/22	T_r_	0.971***		0.952***		0.837***	
	Gs	0.993***	0.973***	0.928***	0.997***	0.852***	0.996***
5/23	T_r_	0.981***		0.756**		0.473	
	Gs	0.989***	0.998***	0.707**	0.997***	0.531	0.998***
5/24	Tr	0.991***		0.861***		0.990***	
	Gs	0.993***	0.999***	0.876***	0.999***	0.992***	0.999***

Note

*, ** and *** indicate that the correlation coefficient tests reach significance levels of 0.05, 0.01, and 0.001, respectively.

The above correlation analysis showed that in comparison to GY5218 and JM325, HN6119 had a better resistance to DHW.

### Test of different resistances to DHW for the three cultivars

To verify the reliability of the above results, an artificially simulated DHW condition experiment was conducted in 2020. Table 6 shows the stress index of the photosynthetic physiological parameters for the three cultivars after DHW stress.

**Table 6:** SI (unit: %) of the different photosynthetic physiological parameters for the three cultivars after DHW stress during the 2020 experiment.

Stress treatment		HN6119			GY5218			JM325		
		P_n_	T_r_	G_s_	P_n_	T_r_	G_s_	P_n_	T_r_	G_s_
Mild	5/23	14	43	54	27	56	66	37	56	65
	5/24	ns	15	17	16	49	35	24	47	37
	5/26	ns	8	16	8	19	32	4	41	54
Severe	5/24	49	50	46	51	45	42	71	56	57
	5/25	26	46	36	31	48	35	40	42	21
	5/27	ns	27	30	21	47	46	29	48	57

Note: ns indicates that *n*_*b*_>*n*_*a*_, and there is no significant impact after the dry hot wind stress.

As shown in Table 6, the variation characteristics of the parameters under mild and severe DHW stress were similar; the SI reached the highest level on the day DHW occurred and then decreased for HN6119. In particular, P_n_ had the highest decreasing speed, and the SI reached the control level on the first day after mild DHW stress and the third day after severe DHW stress. The SI of TKW under mild and severe DHW stress was 0.01% and 0.36%, respectively (shown in Table 7), indicating that HN6119 had a better resistance to DHW. For GY5218 and JM325 under mild and severe DHW stress, P_n_ showed the most significant variation, and the SI had a decreasing trend; however, the SI did not reach the control level on the third day after DHW stress. T_r_ and G_s_ did not show obvious consistent variation after DHW stress. The SI was still at a high level on the third day after DHW stress, indicating that DHW caused damage to the wheat. The SI of TKW under mild and severe DHW stress was 3.51% and 8.12% for GY5218 and 3.57% and 8.84% for JM325, respectively. This result indicated that GY5218 and JM325 had weak resistance to DHW. It can be seen from Table 7 that the effect of DHW on grain morphology (i.e., grain length, grain width, and grain thickness) indicated that the SI of the three cultivars were all smaller under mild DHW stress than under severe DHW stress. At the same time, the SI of grain length was the smallest, and the SI of grain thickness was the largest among the three cultivars. HN6119 had the smallest SI value, and JM325 had the largest SI value among the three cultivars. The above results indicated that the resistance to DHW was different among different cultivars and that DHW had different effects on grain morphology. Therefore, in comparison to the other cultivars, HN6119 had a better resistance ability to DHW.

**Table 7:** TKW (unit: g), grain length (unit: mm), grain width (unit: mm), grain thickness (unit: mm) and SI (unit: %) for the three cultivars under control contrast (CC), mild dry hot wind (M-DHW) stress and severe dry hot wind (S-DHW) stress during the 2020 experiment.

Cultivars		CC	M-DHW	S-DHW	SI % (M-DHW)	SI % (S-DHW)
HN6119	TKW (g)	45.27	45.27	45.11	0.01	0.36
	Grain Length (mm)	6.51	6.5	6.41	0.15	1.54
	Grain Width (mm)	3.02	3.01	2.92	0.33	3.31
	Grain Thickness(mm)	2.42	2.31	2.21	4.55	8.68
GY5218	TKW (g)	43.79	42.26	40.24	3.51	8.12
	Grain Length (mm)	5.61	5.6	5.42	0.18	3.39
	Grain Width (mm)	2.02	2.01	1.91	0.50	5.45
	Grain Thickness(mm)	1.91	1.82	1.71	4.71	10.47
JM325	TKW (g)	48.75	47.01	44.44	3.57	8.84
	Grain Length (mm)	6.81	6.81	6.21	0.00	8.81
	Grain Width (mm)	2.92	2.83	2.51	3.08	14.04
	Grain Thickness(mm)	1.92	1.83	1.61	4.69	16.15

The responses of the three cultivars to DHW were inconsistent under natural and artificial simulation experiments, indicating that the three cultivars possess different DHW resistance abilities.

## Discussion

Under the natural and artificially simulated DHW field experiments, some physiological parameters of the three wheat cultivars, such as photosynthesis, transpiration and stomatal conductance, were analyzed to assess the response differences to DHW of the three cultivars.

In this study, we found that the cultivar with strong resistance to DHW showed better stomatal regulation ability under natural DHW conditions. The transpiration rate and leaf water loss were reduced by stomatal conductance adjustment to avoid serious damage to plants. The physiological functions could be recovered gradually after the end of DHW. The cultivar with weak resistance to DHW showed more leaf water leaves by increasing the stomatal conductance and transpiration rate, and the physiological parameters showed a significant decrease with no increase when the DHW ended. As in the literature [30], the results showed that DHW increased the transpiration intensity and stomatal aperture, which resulted in water loss from leaf cells. This result was consistent with the response of cultivars with weak resistance to DHW in this study. However, the conclusions in previous literature were obtained for one cultivar and did not reveal the response of different cultivars to DHW. Few research results about the recovery of physiological functions after DHW were mentioned in previous literature.

This study shows that the physiological parameters of cultivars with strong resistance to DHW could maintain a significant positive correlation when affected by DHW, which indicates that the cultivars have better self-regulation ability. The physiological parameters of cultivars with weak resistance to DHW showed worse correlations when affected by DHW, especially severe DHW, which means that the regulatory function of wheat was damaged and that normal physiological functions, such as photosynthesis and transpiration, were stressed. However, this finding has not been reported in previous studies [29,41].

According to the analysis, we found that the leaf photosynthetic WUE of the three cultivars was lower when DHW conditions occurred than when no DHW occurred. Because of the cultivar attributes, the reasons for the WUE changes were different. There were two different ways for the decreasing WUE to adapt to adverse conditions: one was to reduce the net photosynthetic rate for the cultivars with better resistance to DHW, and the other was to increase the transpiration rate for the cultivars with relatively weak resistance to DHW.

In this study, the variations in photosynthetic physiological parameters for different cultivars under natural DHW conditions were analyzed. The results show that the P_n_, T_r_ and G_s_ of the three cultivars all decreased on the first day after the end of DHW. Under the artificially simulated DHW conditions, the photosynthetic physiological parameters of different cultivars all decreased compared with those not under DHW conditions, which indicates that DHW has inhibiting effects on the photosynthetic physiological parameters of winter wheat. Zhao et al. [29] reported that the photosynthetic transpiration of wheat flag leaves at the grain filling stage was restrained significantly and caused partial closure of stomata under artificially simulated DHW. A study by Zhang et al. [30] showed that the net photosynthetic rate, transpiration rate, and stomatal conductance of wheat flag leaves on the first day after DHW were lower than those of the control group. These results were consistent with those of our study. Lu et al. [28] reported that the maximum value of stomatal opening of cultivars with no resistance to DHW showed a trend of appearing earlier, its duration was prolonged significantly, and the transpiration intensity had no significant change. The maximum value of stomatal opening of cultivars with strong resistance to DHW showed no significant change, and the transpiration intensity had a significant decreasing trend. There were some differences with the conclusions of this study, which may be related to the difference in cultivar resistance. In short, the three cultivars showed different photosynthetic responses to DHW, reflecting the different resistances of the three cultivars to DHW, as environmental factors such as CO_2_ concentration and soil moisture were consistent in this study.

## Conclusions

The responses of the three winter wheat cultivars to DHW were studied by field experiments under natural DHW conditions. The results suggested that the photosynthetic physiological parameters of P_n_, T_r_, and G_s_ for the three cultivars decreased on the first day after DHW ended; the P_n_, T_r_ and G_s_ of HN6119 recovered with time after DHW; and those of GY5218 and JM325 still decreased. The SI values of TKW under mild and severe DHW stress among the three cultivars were different; HN6119 had the smallest value, GY5218 had the median value, and JM325 had the largest value. The resistance to DHW was different among the three cultivars, and DHW had a different effect on grain morphology. The response differences in the P_n_, T_r_ and G_s_ variation characteristics and their correlations among the three cultivars suggested that HN6119 had a better resistance to DHW, while the resistance of GY5218 and JM325 were relatively weak. Further studies should be carried out to determine how to cope with the negative effect of DHW on crop production under global climate change conditions.

## Supporting information

S1 Data The data used for each figure and table in the manuscript and response to reviewers and the data information. (RAR)

S1 File (XLSX)

S2 File (XLSX)

## References

[1] Smika DE, Shawcroft RW. Preliminary study using a wind tunnel to determine the effect of hot wind on a wheat crop. Field Crops Research. 1980; 3:129–135. 10.1016/0378-4290(80)90018-0.

[2] Coordinated Research Group of Dry Hot Wind for Wheat in North China. Dry hot wind for wheat. Beijing: Meteorological Press, 1988. (in Chinese).

[3] You FC, Hao LS, Shi YS, Duan SL, Kong FC. Causation analysis of dry-hot wind formation in Hebei province winter wheat region. Meteorological monthly. 2007; 33(3):95–100.

[4] Chen XM, Li KJ, Jia YS. Wheat in Hebei. China Agricultural Science and Technology Press. 2008. (in Chinese).

[5] Yang FY, Zhu YJ, Liu WC. Occurrence rules and risk zoning of dry-hot wind in winter wheat producing areas of north China. Journal of natural disasters. 2013; 22(3):112–121. (In Chinese).

[6] Lobell DB, Adam Sibley J. Ortiz-onasterio I. Extreme heat effects on wheat senescence in India. Nature Climate Change. 2012; 2:186–189.

[7] IPCC. Synthesis report of the IPCC fourth assessment report 2007. 2007, http://www.ipcc.ch/publications_and_data/publications_ipcc_fourth_assessment_report_synthesis_report.htm (2013-7-5).

[8] Deng ZY, Zhang Q, Qing JZ. Impact of climate warming and drying on dry-hot wind in the north of China. Journal of Glaciology and Geocryology. 2009; 31(4): 664–671. (In Chinese).

[9] Deng ZY, Wang Q, Zhang Q. Impact of climate warming and drying on food crops in northern China and the countermeasures. Acta Ecologica Sinica. 2010; 30(22):6278–6288. (In Chinese).

[10] Zhang J, Yao F, Li B, Yan H, Hou YY, Cheng GF, et al. Progress in monitoring high-temperature damage to rice through satellite and ground-based optical remote sensing. Science China Earth Sciences. 2011; 54, 1801–1811. (In Chinese) 10.1007/s11430-011-4210-5.

[11] Chen J, Tian Y, Zhang X, Zheng CY, Song ZW, Zhang WJ. Nighttime Warming Will Increase Winter Wheat Yield Through Improving Plant Development and Grain Growth in North China. Journal of Plant Growth Regulation. 2014; 33,397–407. 10.1007/s00344-013-9390-0.

[12] Tavakol A, Rahmani V, Harrington J. Probability of compound climate extreme in a changing climate: A copula-based study of hot, dry windy events in the central United States. Environmental research letters. 2020; 15, 104058. 10.1088/1748-93261/abblef.

[13] Liu EK, Mei XR, Yan CR, Gong DZ, Zhang YQ. Effects of water stress on photosynthetic characteristics, dry matter translocation and WUE in two winter wheat genotypes. Agricultural Water Management. 2016; 167(31):75–85.

[14] Chen XG, Tian GP, Qin ZL, Bi X. High Daytime and Nighttime Temperatures Exert Large and Opposing Impacts on Winter Wheat. Weather, Climate, and Society. 2019; 11(4):777–790. 10.1175/WCAS-D-19-0026.1.

[15] Wang S, Zheng H, Liu SH, Miao YC, Li J. Numerical study on the stomatal responses to dry-hot wind episodes and its effects on land-atmosphere interactions. PloS ONE. 2016; 11(9): e0162852. doi: 10.1371/journal.pone.0162852 27648943 PMC5029913

[16] Zhao JF, Zhao YX, Guo JP, Fang SB. Spatial-temporal changes of dry-hot wind for winter wheat in Huanghuaihai Plain during the past 50 years. Scientia agricultura sinica. 2012; 45(14):2815–2825. (In Chinese) doi: 10.3684/j.issn.0578-1752.2012.14.004,

[17] Lydolph PE. The Russian Sukhovey. Annals of the association of American geographers. 1964; 54(3):291–309. 10.1111/j.1467-8306.1964.tb00490.x.

[18] Lydolph PE. Williams TB. The North American sukhovey. Annals of the association of American geographers. 1982; 72:224–236. 10.1111/j.1467-8306.1982.tb01821.x.

[19] Tavakol A, Vahid R, John H. Temporal and spatial variations in the frequency of compound hot, dry, and windy events in the central United States. Scientific Reports. 2020; 10:15691. doi: 10.1038/s41598-020-72624-0 32973168 PMC7515889

[20] Huo ZG, Shang Y, Wu DR. Review on disaster of hot dry wind for wheat in China. Journal of Applied Meteorological Science. 2019; 30(2):129–141. (In Chinese) doi: 10.11898/1001-7313.20190201

[21] Coordinated Research Group of Hot-arid Wind for Wheat in North China. Study on the meteorological index of hot-arid wind for wheat. Scientia Agricultura Sinica. 1983; (4): 68–75. (In Chinese).

[22] Coordinated Research Group of Hot-Dry Wind for Wheat in North China. Effect of dry-hot wind on grain filling rate of Wheat. Meteorological Monthly. 1983; 9(5):22–24. (In Chinese).

[23] The Cooperated Research Group on Dry-hot-wind Injury in Wheat in Thirteen Provinces and Municipalities in North China. Study on the injurious mechanism of hot weather with dry wind (HDW) in Wheat. Acta Agronomica Sinica. 1984; 10(2):105–112. (In Chinese).

[24] Wu DR, Liu JD, Liu L, Jiang CY, Xu YL. Spatiotemporal distribution characteristics of dry hot windy days in North China Plain in recent 50 years. Journal of natural disasters. 2012; 21(5):167–172. (In Chinese).

[25] Wang CY, Pan YR, Ji GS. Index analysis of dry hot wind year’s type and forecasting model in Shijiazhuang district. Acta Meteorological sinica. 1991; 49(1):104–107. (In Chinese).

[26] Li S, Zhang L, Huang BX, He L, Zhao JF, Guo AH. A comprehensive index for assessing regional dry-hot wind events in Huang-Huai-Hai region, China. Physics and chemistry of the earth. 2020; 116. 10.1016/j.pce.2020.102860.

[27] Gong SX. A preliminary study on the resistance to the hot-dry wind of the various types of wheat varieties. Acta Agriculturae Universitatis Pekinensis. 1981; 7(3): 89–98. (In Chinese).

[28] Lu ZD, Chang SJ, Liu XZ, Guo XQ. Study on the resistibility of different wheat varieties. Inner Mongolia agricultural science and technology. 1983; 6(10):20–25. (In Chinese).

[29] Zhao F.H.; Ju H.; Ou Y.Z. Effects of Dry-hot wind on photosynthesis and transpiration of flag leaf of winter wheat at filling stage. Acta Agriculturae Boreali-Sinica, 2013, 28(5):144–148(In Chinese).

[30] Zhang ZH, Cheng L, Li SL. Dry-hot wind effects on physiology of winter wheat. Chinese Journal of Ecology. 2015; 34(3): 712–717. (In Chinese).

[31] Zhu KY, Sun ZG, Zhao FH, Yang T, Tian ZR, Lai JB, et al. Remotely sensed canopy resistance model for analyzing the stomatal behavior of environmentally-stressed winter wheat. ISPRS Journal of Photogrammetry and Remote Sensing. 2020; 168:197–207.

[32] Zhao FH, Zhu KY, Long BJ, Tian ZR, Lai BJ, Sun ZG. Effect of brackish water irrigation on the resistibility of winter wheat leaf to dry-hot wind. Chinese Journal of Eco-Agriculture. 2020; 28(10):1609–1617. doi: 10.13930/j.cnki.cjea.200077

[33] Huo ZG, Jiang Y, Li SK. Disaster grade of dry hot wind for wheat. Beijing: Meteorlolgical Press. 2007. (In Chinese).

[34] Evans LT. Crop physiology. Cambridge: Cambridge University Press, 1975; 01–150.

[35] Chen Y, Xu DQ. Two patterns of leaf photosynthetic response to irradiance transition from saturating to limit ingone in some plant species. New Phytol. 2006; 169:789–798. doi: 10.1111/j.1469-8137.2005.01624.x 16441759

[36] Xu DQ, Shen YK. External and internal factors responsible for midday depression of photosynthesis. In: Pessarakli M (ed). Handbook of Photosynthesis. 2-nd. Boca Raton: CRC Press, Taylor & Francis Group, 2005; 287–297.

[37] Xu DQ. Some noteworthy problems in measurement and investigation of photosynthesis. Plant Physiol Commun. 2006; 42(6):1163–1167.

[38] Xu DQ, Li DY, Shen YG. Study on the "siesta" phenomenon of wheat leaf photosynthesis in the field. Acta Phytophysiologica Sinica. 1984; 10(3):269–275. (In Chinese).

[39] Chen GY, Chen J, Xu DQ. Thinking about the relationship between net photosynthetic rate and intercellular CO_2_ concentration. Plant Physiology Communications. 2010; 46(1):64–66. (In Chinese).

[40] Farquhar GD, Sharkey TD. Stomatal conductance and photosynthesis. Annual Review of Plant Physiology. 1982; 33:317–345.

[41] Yin YF, Zhang CL, Yao FX. Relationship of Leaf Photosynthetic Rate with Stomatal Resistance and Stomatal Conductance to CO_2_ in Wheat Cultivars. Acta Agronomica Sinica. 1995; 21(5): 561–567. (In Chinese).

